# Pharmacogenetics of new classes of antidiabetic drugs

**DOI:** 10.17305/bjbms.2021.5646

**Published:** 2021-12

**Authors:** Selma Imamović Kadrić, Aida Kulo Ćesić, Tanja Dujić

**Affiliations:** 1 Department of Biochemistry and Clinical Analysis, Faculty of Pharmacy, University of Sarajevo, Sarajevo, Bosnia and Herzegovina; 2 Department of Pharmacology, Clinical Pharmacology and Toxicology, Faculty of Medicine, University of Sarajevo, Sarajevo, Bosnia and Herzegovina

**Keywords:** Type 2 diabetes, pharmacogenetics, personalized medicine, new antidiabetics

## Abstract

Type 2 diabetes (T2D) has a continuously rising prevalence worldwide. Pharmacogenetics has been recognized as a promising concept for pharmacological treatment of T2D, as antidiabetic drugs are not equally effective and safe for all patients, and the costs of diabetes treatment are increasing. The latest published guidelines on T2D treatment firmly endorse the use of newer antidiabetic drugs, sodium-glucose cotransporter-2 inhibitors (SGLT2i), dipeptidyl peptidase-4 inhibitors (DPP-IVi), and glucagon-like peptide-1 receptor agonists (GLP-1RA), considering their satisfactory pharmacological effect and good safety profile. Furthermore, SGLT2i and GLP-1RA show protective effects in patients with established atherosclerotic cardiovascular disease and chronic kidney disease. However, there has been growing evidence that the effectiveness and safety of these drug classes could depend on genetic variability. Here, we summarized the results of the published studies on the pharmacogenetic biomarkers for the three drug classes. A number of genetic variations have been investigated so far. The explored candidate genes mostly encode drug targets, drug-metabolizing enzymes, and genes linked to T2D risk. Although many of the results are promising, it is still necessary to obtain more information from larger controlled studies to confirm their clinical significance. This approach may lead towards more personalized treatment for patients with T2D.

## INTRODUCTION

Diabetes is a serious long-lasting pathological state characterized by an inability of a body to carry out the physiological role of insulin. With currently more than 463 million people suffering from diabetes, the number is expected to reach 578 million by 2030, and 700 million by 2045 [1]. It is known now that the impaired interplay between beta cells in pancreas and insulin-sensitive tissues leads to the development of the most common form of the disease, Type 2 diabetes (T2D) [2].

The latest published consensus report of the American Diabetes Association (ADA) and the European Association for the Study of Diabetes (EASD) suggests a choice between five antidiabetic drug groups as the second line therapy for T2D. Three of these are the newer antidiabetic drug classes: sodium-glucose cotransporter-2 inhibitors (SGLT2i), dipeptidyl peptidase-4 inhibitors (DPP-IVi), and glucagon-like peptide-1 receptor agonists (GLP-1RA) [3].

Precision medicine is a newer therapeutic concept which strives to distinguish patients based on their treatment response [4]. Pharmacogenetics is a tool of precision medicine that enables the determination of an optimal pharmacological agent for a single patient according to genetic traits [5].

**Figure 1:**
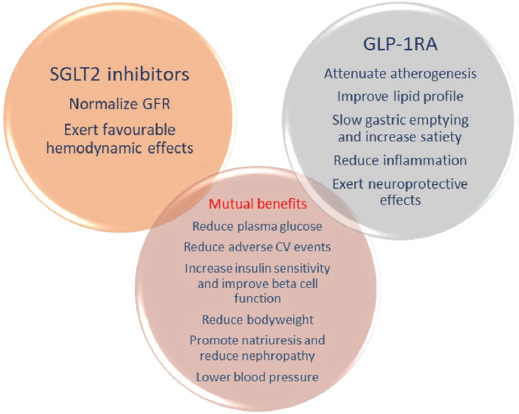
Benefits of treatment with SGLT2i and GLP-1RA based on their direct and indirect metabolic, cardiovascular, and renal effects [10]. SGLT2i: Sodium-glucose cotransporter-2 inhibitors, GLP-1RA: Glucagon-like peptide-1 receptor agonists.

It is evident today that antidiabetic drugs are not equally effective and safe for all patients and the costs of diabetes treatment are still increasing. However, technological tools that support the implementation of pharmacogenetics are rapidly developing [6]. Therefore, it is appealing to turn towards the individualized pharmacologic approach to treat T2D and its complications [7,8]. The insight into the pharmacogenetics of the three mentioned antidiabetic drug groups is of special importance. SGLT2i and GLP-1RA are gaining special attention since the most recent ADA-EASD guidelines endorse their use in patients with the diagnosis of atherosclerotic cardiovascular disease (ASCVD) and chronic kidney disease (CKD), apart from the other benefits [3,9-11] (Figure 1). However, there has been growing pharmacogenetic evidence that the effectiveness and safety of these drug classes rely to a certain extent on variations in candidate genes [12]. Thus, by searching the PubMed database with the keywords: “Pharmacogenetics, Type 2 diabetes”, “SGLT-2 inhibitors pharmacogenetics”, “DPP-IV inhibitors pharmacogenetics”, “GLP-1R agonists pharmacogenetics”, and “Type 2 diabetes personalized medicine”, the aim of this review was to summarize the results of the studies published so far on the potential use of pharmacogenetics in the treatment with newer antidiabetic drugs. We also searched the lists of the references in the primary articles to retrieve the additional articles of interest. All articles that matched the keywords, published before September 2020, were considered.

## SGLT2 INHIBITORS (GLIFLOZINS)

Gliflozins act by inhibiting the Type 2 of high-affinity SGLTs, which are responsible for glucose reabsorption from the renal proximal tubule, leading to glycosuria [13-16]. Although most of gliflozins that are currently used also inhibit SGLT1, they show notably higher potency for SGLT-2 over SGLT-1 (~260:1 for canagliflozin and ~2700:1 for empagliflozin), which is why they are sometimes marked as “selective” SGLT2 inhibitors [15]. The inhibition of the SGTL2 transporter results in a reduction in fasting plasma glucose of 1.3–2.4 mmol/L and reduction in HbA1c levels of 0.4–1.1% [17]. Still, gliflozins show a low risk of hypoglycemia since they exert their effect independently of insulin [18]. Apart from the most common side effects which include mild genital infections, urinary tract infections, and volume related adverse effects, their safety profile is considered satisfactory [19]. Importantly, SGLT2i show clinically relevant protective effect not only in T2D patients with CKD but also in non-diabetic population [20].

There is high inter-individual variability in response to the treatment with SGLT2 inhibitors. Several studies investigated the role of genetics in efficacy of these medications. Type 2 of SGLT transporter is encoded by *SCL5A2* gene located on the chromosome 16p11.2 [21]. Initial pharmacogenetic studies related to SGLT2 inhibitors explored mutations in this gene since they have been responsible for Familial renal glycosuria (FRG). This rare hereditary kidney disease is typically characterized by reduced urinary glucose reabsorption which leads to chronic glycosuria [22]. To date, more than 70 *SLC5A2* gene mutations have been identified and correlated with FRG [23,24].

The first study that explored the role of genetic variations in *SLC5A2* gene was conducted by Enigk et al. [25]. The authors investigated the association between four common SNPs in the SGLT2 gene region, namely, rs9934336, rs3813007, rs3813008, and rs3116150 with glucose parameters and BMI in people without diabetes as well as association with T2D. The main subject group consisted of 1013 Eastern Germans of which 106 subjects were diagnosed with T2D. The control group consisted of 2042 individuals with 359 patients with T2D. The study did not find any association between investigated genetic variants and T2D traits. However, the rs9934336 SNP had a nominally significant effect on glucose concentrations and insulin levels in individuals without diabetes in both the main and the control group. Namely, the AA genotype of rs9934336 was associated with reduced glucose concentrations after 30 minutes and insulin levels after 120 minutes during an oral glucose tolerance test (OGTT). Two other SNPs, rs3813008 and rs3813007, were significantly associated with insulin and glucose levels, respectively, after 30 minutes in the OGTT, in the main group. Tested variants did not show the effect on the beta cell function or insulin resistance in any study group. This was the first study to investigate the effects of variants in *SLC5A2* gene on glucose homeostasis in people without T2D. Results of the study implicate that potentially inactivating variants in *SLC5A2* gene imitate SGLT2 inhibition and cause lower glucose and insulin levels. Moreover, the results of the study were consistent with the earlier results of studies in animals that suggested that the effects of SGLT2 inhibitors are more prominent in animals with diabetes [26]. In addition, animal studies show that deletion of the SGLT2 gene results in significantly preserved beta cell function and decreases the incidence of beta cell death [27].

Zimdahl et al. have studied the effects of five common SNPs (rs9934336 G>A, rs3813008 G>A, rs3116150 G>A, rs3116149 G>A, and rs11646054 G>C) in the *SLC5A2* locus on metabolic traits in individuals at risk for T2D. Three out of five SNPs were investigated in the earlier study by Enigk et al. In addition, they explored the pharmacogenetic effects of the SNPs in patients treated with empagliflozin. They included a total of 2600 individuals in the cross-sectional study and 908 patients in the pharmacogenetic study. The study did not find any significant association between SNPs and either with tested metabolic parameters or with empagliflozin treatment response assessed as difference in HbA1c and fasting glucose after 24 weeks of follow-up [28].

A recent study done by Drexel et al. [29] investigated the association between genetic variants in the *SLC5A2* gene with T2D and the risk of cardiovascular disease. A total of 1684 patients with risk of coronary artery disease (CAD) subjected to coronary angiography were genotyped for tagging SNPs rs9934336, rs3813008, and rs3116150, in the SGLT2 gene region. A total of 400 patients had T2D. The authors confirmed results from the previous study by Enigk et al. – the minor A allele in rs9934336 SNP was linked to decreased HbA1c, decreased fasting glucose and 120 minutes’ glucose values during OGTT. However, more importantly, this study found a significant association between the A allele of rs9934336 and reduced risk of T2D in both univariate and multivariate logistic regression statistical model, adjusted for sex, age, BMI, the presence of metabolic syndrome, and hypertension. No association was found between *SLC5A2* SNPs and the risk of CAD. In addition, the authors performed a meta-analysis including results from the two previous studies [25,28]. Interestingly, by combining the studies’ results they confirmed that the minor allele of rs9934336 was significantly associated with reduced risk of T2D, although previous individual studies failed to demonstrate such association. In general, differences in patient characteristics and genetic background between separate studies could have led to different observations. Nevertheless, it is plausible that reduced function of SGLT2 can prevent hyperglycemia that, in the long-term, can protect from the development of T2D in an individual. Because of the noticed protective effect of rs9934336, but also the paucity of evidence regarding pharmacological intervention with gliflozins in the setting of different genotypes for rs9934336, it would be pertinent to design further studies to explore potential pharmacogenetic aspect of this variant.

A small retrospective study was recently conducted to test the association between a polymorphism in the *SLC5A2* gene and development of macro- and microvascular complications in Slovenian patients with T2D [30]. A total of 181 patients with T2D were genotyped for *SLC5A2* rs9934336 G>A polymorphism and monitored for kidney function and diabetic retinopathy. Contrary to the previous research results [25,28], the study found that the carriers of at least one minor (A) allele of *SLC5A2* rs9934336 had increased levels of fasting blood glucose and HbA1c. Furthermore, they found that *SLC5A2* rs9934336 G>A polymorphism is significantly associated with the risk of diabetic retinopathy. The explanation might be drawn from animal studies’ data which showed that SGLT2 is also expressed in retinal pericytes where it controls glucose entering and therefore retinal energy metabolism [31,32]. Genetic variations in the SGLT2 gene might thus alter the pericytes morphology and function and finally lead to complications.

The other obvious pharmacogenetic candidates are genes encoding enzymes responsible for the metabolism of gliflozins. This drug class is extensively metabolized in the liver, and glucuronidation is the main metabolic pathway [33]. There are only two studies published on the genetics of the UGT enzymes in relation to treatment with gliflozins, though there is a lack of evidence from typical pharmacogenetic studies. A study done by Francke et al. in 2015 [34] aimed to determine the main UGT enzymes responsible for the metabolism of canagliflozin. UGT1A9 and UGT2B4 were identified as the main enzymes that produce the two substantial O-glucuronide metabolites of canagliflozin *in vitro*. Moreover, since the genes encoding UGT enzymes are genetically polymorphic, a pool pharmacogenomic analysis on 134 participants’ (mainly Caucasians) samples has been conducted [34]. Carriers of the UGT1A9*3 allele (rs72551330 T>C; p.Met33Thr), which exhibit a reduction in glucuronidation rate, had higher canagliflozin plasma exposure compared to non-carriers (C_max,ss_ 11%; AUCŢ,ss 45% higher). In contrast, plasma concentrations of canagliflozin were not different between carriers of the wild-type allele and variant allele of UGT2B4 (UGT2B4*2), in spite of the latter having reduced levels of O-glucuronide metabolites. Nonetheless, the observed differences in canagliflozin concentrations in different genotype groups for UGT1A9*3 are not considered clinically relevant. Furthermore, there was no increase in overall adverse effects incidence in the carriers of the minor allele and they presented a rather small subgroup in the explored population. Furthermore, although the effect of UGT2B4*2 genotype on altered metabolism of canagliflozin cannot be neglected, it is likely to be small.

The other research performed by Hoeben et al. [35] also focused on canagliflozin. It was based on the development and evaluation of a mathematical population pharmacokinetic model that analyzed data from healthy volunteers and patients with T2D included in Phases I, II, and III trials. Different covariates were used to understand canagliflozin pharmacokinetics, detect statistically significant covariates in the model, and finally predict their clinical relevance. A total of 1616 subjects were included to gain relevant pharmacokinetic information. The polymorphism of the UGT1A9 gene (UGT1A9*3; rs72551330 T>C; p.Met33Thr) was added as a covariate since it could directly influence canagliflozin pharmacokinetics. It was noticed that carriers of the UGT1A9*3 allele had greater exposure to canagliflozin (dose-normalized AUC was 26% higher). Again, this subgroup of patients was small, so the model did not recognize the genetic polymorphism in the UGT enzyme as a factor requiring dose adjustment during canagliflozin therapy.

Obviously, there is not much evidence on the pharmacogenetics of gliflozin pharmacotherapy. However, since SGLT2 inhibitors are a promising option in T2D treatment, it is of great importance to further target their pharmacogenetic aspects using properly-sized cohorts with adequate study designs [21]. The pharmacogenetic studies of SGLT2 inhibitors are summarized in Table 1.

**TABLE 1:**
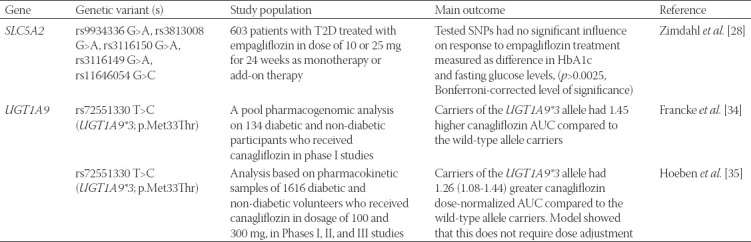
Pharmacogenetic studies of SGLT2 inhibitors

## DPP-IV INHIBITORS (GLIPTINS)

Gliptins were first approved in 2006 when sitagliptin was introduced. Most of the drugs from this class are orally administered once or twice daily and are quickly absorbed.

Gliptins have a low potential for drug-drug interactions as they do not significantly induce or inhibit CYP enzymes nor they substantially attach to plasma proteins. Only saxagliptin is metabolized by CYP3A4/5. Other gliptins are usually excreted by kidneys except for linagliptin which is excreted by bile [36]. DPP-IV inhibitors act to re-establish the incretin effect which is impaired in patients with T2D, by targeting the enzyme dipeptidyl peptidase DPP-IV. This prevents quick degradation of the two incretin hormones, glucagon-like peptide-1 (GLP-1) and glucose-dependent insulinotropic polypeptide (GIP) and preserves physiological levels of glucose. Inhibition of DPP-IV by gliptins results in two-three-fold elevation of endogenous incretins [37]. Furthermore, gliptins exert a body weight neutral effect [38]. The expected efficacy of gliptins in regard to reducing HbA1c is rather modest, 0.5-0.8%, and is dependent on HbA1c baseline level [39]. Patients usually tolerate well treatment with these medications [40]. A meta-analysis from 2012 suggests that DPP-IV inhibitors are safe with regard to long-term use [41].

Pharmacogenetic aspects of incretin mimetics (DPP-IVi and GLP-1RA), as relatively recent drug classes, are so far sparingly explored with conclusions from the studies rather inconsistent [42]. Still, it is evident that response to DPP-IV inhibitors varies greatly among individuals and it is reasonable to suspect that genetics plays a role in it. Gliptins are not extensively metabolized in the liver and thus genetic variations in the metabolic enzymes or hepatic drug transporters cannot be used as pharmacogenetic targets [43]. Therefore, it is appealing to investigate the effects of genetic variants in incretin receptors or in the genes that were previously connected to T2D or risk of T2D. Furthermore, other genes that might be linked to incretin mimetics response are discovered usually through genome wide association studies (GWAS) [44,45].

### Genes Coding Drug Targets of Gliptins

Since the mechanism of the pharmacological action of DPP-IV inhibitors is linked to the GLP-1R [46,47], the GLP-1R gene was a plausible target to explore. In an observational study of 264 patients with T2D on gliptin treatment for 24 weeks, the effect of rs3765467 SNP (G>A; p.Arg131Gln) in GLP-1R was examined. The results suggest that this genetic variation in GLP-1R gene could influence the efficacy of DPP-IV inhibitors treatment. Patients who carried at least one minor allele (GA or AA genotype) had greater HbA1c reductions after treatment with DPP-IV inhibitors compared to homozygous wild-type allele carriers (GG). The difference was significant also in the multivariate analysis. However, due to the observational study design, the authors could not evaluate the effects of other factors that could affect glycemic status in the participants. Furthermore, these conclusions applied to Asians in which this SNP is relatively more frequently present compared to other populations [48].

One study investigated another polymorphism within GLPR-1 gene in relation to therapy with gliptins [49]. The GLP1R rs6923761 (Gly168Ser) and GIPR rs10423928 (T>A) variants were genotyped in 140 patients with T2D. In addition to GLP-1R, the GIP-R is also the indirect target of gliptin pharmacological action. The main outcome evaluated was the reduction in HbA1c levels after 6 months of treatment initiation. The results showed that the variant in the GLP-1R gene significantly affects therapy outcome. Carriers of the minor allele had substantially lower HbA1c reduction compared to the wild-type allele carriers. According to the findings of *in vitro* studies, the mechanism of this reduced effect might be reduced expression of the receptor or decreased intracellular Ca^2+^ mobilization. This could further reduce GLP-1 stimulated insulin secretion and thus explain the reduced gliptin effect [50]. It is important to note that the observed genotype-dependent effect was equal to the average effect of this drug class and thus this variant might represent a promising pharmacogenetic candidate [49].

Wilson et al. published the study which investigated the influence of genetic variability in the DPP-IV gene on the activity of DDP-IV enzyme during sitagliptin treatment [51]. In a double-blind, crossover fashion, 27 patients with T2D and 38 healthy controls were randomized to receive a single dose of 200 mg sitagliptin or repeated 100 mg dose of sitagliptin for 4 or 7 days or matching placebo. In a multivariate analysis, among other factors, the authors found that the genotype of rs2909451 C>T variant in the DPP-IV gene was a predictor of DPP-IV activity during treatment with sitagliptin. Namely, rs2909451 TT genotype carriers had greater DPP-IV activity while they were on sitagliptin treatment. However, small sample size and no data from similar replication studies do not permit any definite conclusions regarding this finding.

Although it would be interesting to investigate the role of genetic variants in genes encoding other physiological DPP-4 substrates, such as peptide tyrosine tyrosine (PYY), in DPP-IV response, no pharmacogenetic studies on this have been conducted.

### Genes linked to T2D

One of the earliest studies that tackled genetics and gliptins’ treatment was done by t Hart et al. [8,52]. The study was based on earlier findings showing that GLP-1 induced insulin secretion is influenced by genetic variants [53]. The authors used fine-mapping approach to detect potential variants in non-diabetic subjects, followed by testing the effects of the detected SNPs on the treatment with gliptins in a total of 354 patients with T2D. The results showed that the G allele of the rs7202877 T>G SNP near CTRB1/2 was associated with the reduced effect of DPP-IV inhibitors. The carriers of the G allele had 0.51 ± 0.16% lower HbA1c response compared to the TT carriers. The minor G allele of the rs7202877 has been associated earlier with the protective role in T2D development [54]. This is in line with the increased GLP-1 stimulated insulin secretion in the G allele carriers [52]. Nevertheless, further well-powered studies are warranted to confirm and explain this pharmacogenetic finding.

CDK5 regulatory subunit associated protein 1-like 1 (CDKAL1) gene encodes enzyme from the methylthiotransferase family. Its exact role is still unknown; however, the polymorphisms of *CDKAL1* gene are associated with the increased T2D risk, probably due to impaired insulin secretion [55]. Osada et al. investigated whether polymorphisms in the *CDKAL1* gene (rs7754840 G>C and rs756992 A>G) could influence therapeutic response to anti-diabetes agents [56]. They analyzed medical records of 798 patients with T2D to test if CDKAL1 genotype can influence therapeutic response to the following drug classes: biguanides, sulfonylureas, DPP-IV inhibitors, thiazolidinediones, glinides, and GLP-1RA. Interestingly, the study found significant differences in HbA1c reduction among genotypes only for DPP-IV inhibitors. Patients who carried T2D risk alleles (C for rs7754840 and G for rs756992) had significantly greater HbA1c reduction after 3 months of treatment with DPP-IV inhibitors. The authors suggested that the mechanistic explanation may be the stimulation of insulin secretion by DPP-IV inhibitors, which might substitute the reduced ATP level in risk variant carriers, and thus lead to better response [56].

The *KCNJ11* gene plays a role in insulin secretion by encoding a subunit of pancreatic ATP-dependent potassium channel. Since incretin mimetics are insulin-stimulating drug classes, this gene may have importance as a part of the insulin signaling pathway. In a study that aimed to detect clinical and genetic factors that affect gliptin treatment response, variants rs2285676 C>T, rs5218 G>A, and rs5210 G>A in the *KCNJ11* gene were genotyped based on their earlier established link to diabetes. The study included 662 patients with T2D, of whom 331 patients were treated with DPP-IV inhibitor and 331 patients with another antidiabetic drug. The treatment response was evaluated as on-treatment HbA1c equal to or lower than 7.0%. Authors found that the carriers of the *KCNJ11* rs2285676 CC genotype had a 2-time higher chance of responding to gliptin treatment compared to other patients [57].

The *KCNQ1* gene encodes a subunit of a ubiquitous voltage-gated potassium channel. It is expressed also in pancreatic beta cells and has a role in insulin secretion [58]. The *KCNQ1* variants have been linked to the T2D risk [59,60]. A pilot pharmacogenetic study examined the influence of rs163184 T>G variant in this gene on the glycemic response to gliptin treatment in 137 patients. Sitagliptin or vildagliptin in a daily dose of 100 mg was added to metformin or metformin/sulfonylurea therapy. The patients were followed for 6 months. The study found the association of the minor G allele with poorer response to drugs from this class, namely, a smaller reduction in HbA1c. The observed difference between the TT and GG genotypes was 0.6% which could be clinically relevant if confirmed in larger cohorts [61].

Insulin secretion pathway also includes G-protein coupled receptor 40 (GPR40) which is regulated through activation of protein kinase D 1 (PRKD1), a serine/threonine kinase. The impaired function of PRKD1 could affect beta cell insulin secretion and possibly gliptins efficacy [62,63]. In a GWAS study, 171 Taiwanese patients treated with DPP-IV inhibitors longer than 60 days, mostly as an add-on to other antidiabetic drugs, were included in the study. Patients were divided into response sensitive and resistant groups based on the change in HbA1c levels. A variant in the intron region of the *PRKD1* gene (rs57803087 A>G) was significantly associated with gliptin therapeutic response in T2D. This finding supports the hypothesis that variants in the genes controlling beta cell function can affect the efficacy of DPP-IV inhibitors. However, this study had limited statistical power [63].

The *TCF7L2* gene rs7903146 C>T variant has been repeatedly and strongly connected to T2D risk [64-66], although the exact mechanism is not fully understood. It has been associated with proliferation and function of beta cells, insulin synthesis and secretion, and modulation of incretin action [67]. Interestingly, the rs7903146 T allele has been linked to impaired incretin-stimulated insulin secretion in some [68-70], but not all studies [71]. The impact of the rs7903146 C>T variant on the response to treatment with linagliptin was evaluated in 961 patients with T2D followed for 2 years [72]. No significant differences were observed between homozygous and heterozygous carriers of the wild-type allele. However, the homozygous carriers of the minor allele (TT) had significantly reduced response measured as HbA1c reduction compared to patients with the CC genotype. Although the clinical significance of this association is yet to be proved, these results contribute to the role of T2D risk-genes in gliptin therapeutic response.

An interesting study has been conducted by Matsui et al. [73] which investigated variants in the human Interleukin-6 (IL-6) gene in relation to DPP-IV inhibitors response. IL-6 from muscle cells promotes GLP-1 secretion in animal models, similarly to gliptins. A total of 316 Japanese patients were genotyped for rs1800796 (G>C) and rs2097677 (G>A) variants and followed for 3 or 4 months after initiation of gliptin therapy. The response was defined as achieving HbA1c reduction of more than 0.2%. In a multivariate analysis, it was shown that the rs1800796 G/*-rs2097677 A/* diplotype confers a reduced risk of not responding to DPP-IV inhibitors compared to the C/C-G/G diplotype, in patients who had moderate/high level of physical activity during the treatment. Further studies are required to confirm these results and explain the mechanism of this possible relationship.

Finally, since it is known from *in vitro* data that gliptins show protective effects against hepatic steatosis [74], Kan et al. attempted to link patatin-like phospholipase 3 gene (*PNPLA3*) rs738409 C>G genotype with the efficacy of alogliptin in NAFLD patients with T2D [75]. They genotyped 41 T2D patients with established NAFLD and evaluated their clinical data retrospectively before and after the treatment with alogliptin. Statistically significant positive correlations between improvement in HbA1c and changes in AST and ALT levels were detected only in the carriers of the risk G allele. In addition, in the weight loss group, the G allele carriers showed higher decrease in the levels of total cholesterol, triglycerides and hyaluronic acid. Therefore, the authors concluded that *PNPLA3* rs738409 genotype could influence the therapeutic efficacy of alogliptin in the amelioration of NAFLD.

A summary of pharmacogenetic studies of DPP-IV inhibitors is given in Table 2.

**TABLE 2:**
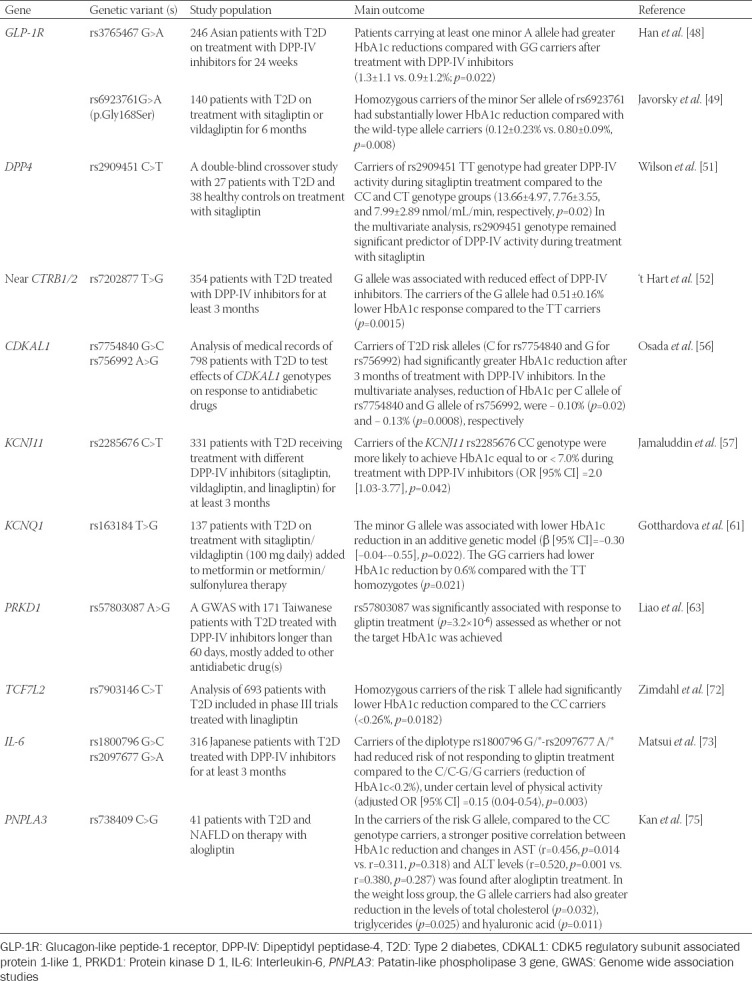
Pharmacogenetic studies of DPP-IV inhibitors

### GLP-1 receptor agonists

The GLP-1RA were designed to imitate GLP-1 activity with structures modified to resist quick metabolic degradation. At present, there are six GLP-1RA approved for clinical use as the subcutaneous formulations [76]. They lower blood glucose levels by increasing pancreatic insulin secretion and by suppressing the secretion of glucagon in a glucose-dependent manner [77]. Controlled studies have shown that GLP-1RA are highly efficacious, exert minimal risk of hypoglycemia, and promote body weight loss [78]. Overall GLP-1RA are considered safe antidiabetic drugs in patients who cannot use metformin or when it is insufficient [79]. The most common adverse effects are gastrointestinal [80].

Studies have shown that long-acting liraglutide and semaglutide have cardioprotective benefits [81]. Interestingly, a large cohort study showed that genetics can have a role in this protective effect. Namely, rs10305492 variant in the *GLP1R* gene was linked to a lower risk of heart disease, but also with the lower fasting glucose and reduced T2D risk [82]. In addition, two other more recent studies identified genetic variants that were associated with the CAD risk. A GWAS identified a variant rs57922 (C/C genotype) linked to higher GLP-1 secretion and CV benefits from intensive hypoglycemic treatment [83]. Furthermore, among 11 tested tagging SNPs in the *GLP1R* gene in the Chinese Han population with T2D, it was shown that carriers of the GG genotype of rs4714210 variant had lower CV risk compared to the AA carriers [84].

Several studies explored the impact of genetic variations on the pharmacological effect of GLP-1RA. Beinborn et al. were the first who discovered *in vitro* that a naturally occurring variant in GLP-1R causes a significant reduction in receptor function, and also reduced agonist responsiveness [85]. More recently Lin et al. tried to explain unresponsiveness to treatment with GLP-1RA and link it to the genetic variability in the GLP-1R gene. They have tested the hypothesis in 36 poorly controlled patients with T2D who were genotyped for 13 different variations. Patients received exenatide for 3 days after receiving subcutaneous insulin infusions for 6 days. Although authors found a significant association of variants rs3765467 C>T and rs761386 C>T with the change in the standard deviation of plasma glucose levels during exenatide treatment, it became insignificant after multivariate analysis [86]. Yu et al. also followed patients with T2D for response to exenatide treatment. A total of 285 overweight Chinese patients with T2D were recruited and genotyped for two common variants rs3765467 C>T and rs10305420 (C>T; p.Pro7Leu). The treatment outcome was measured as the reduction in HbA1c and BMI after 6 months of treatment. The study found that the minor allele of rs10305420 was consistently associated with a decreased reduction in body weight and HbA1c during exenatide treatment, thus making this variant a potentially good pharmacogenetic marker especially in overweight diabetic patients [87]. Interestingly, one earlier published study investigated the same SNP (rs10305420 C>T; p.Pro7Leu) in obese women with polycystic ovary syndrome. They also found that carriers of the minor allele had a poorer response to liraglutide in terms of weight loss. Namely, they lost <5% of their initial body weight [88]. Another study explored pharmacogenetic aspects of liraglutide effects on metabolic traits and weight loss in relation to rs6923761 (G>A; p.Gly168Ser) polymorphism. It showed that the variant affected anthropometric parameters in overweight patients with T2D. The minor A allele was associated with a greater reduction in BMI, weight, and fat mass during liraglutide treatment [89].

Similarly, a recent study explored whether liraglutide-driven prolongation of gastric emptying and weight loss are associated with GLP1R or TCF7L2 genetic variants. It was found that the minor A allele of rs6923761 variant was associated with slower gastric emptying after liraglutide or exenatide treatment in obese individuals; however, it did not affect weight loss significantly [90]. Another recent study tested the association of 27 tagging SNPs in the *GLP1R* locus with gastric emptying rate variability in healthy volunteers. They found a significant effect of rs742764, rs2254336, rs9283907, rs2268657, and rs2254336 variants on gastric emptying rate; however, pharmacogenetic aspects were not investigated [91].

De Luis et al. investigated the effect of a common rs1049353 (G>A) variant in the cannabinoid receptor 1 (CNR1) gene on treatment response in obese patients with T2D [92]. Genetic variants in the *CNR1* gene are likely associated with variability in body weight and energy balance [93,94]. Anthropometric and metabolic parameters were assessed at baseline and after 14 weeks of treatment with liraglutide. Although all patients lost weight during the treatment, only carriers of the minor rs1049353 A allele showed an improvement in insulin resistance, whereas GG carriers had lower cholesterol levels after weight loss.

Several other studies have been conducted to investigate the pharmacogenetics of exenatide. Zhou et al. followed 101 newly diagnosed patients with T2D for 48 weeks during treatment with exenatide [95]. They measured glycemic and beta cell function parameters, including fasting proinsulin/insulin (PI/I) ratio, in all patients. The patients were genotyped for rs1416406 variant in sortilin-related VPS10 domain-containing receptor 1 gene (*SORCS1*). The previous studies indicated that *SORCS1* gene is linked to T2D risk through impaired insulin secretion, as well as obesity [96,97]. All patients showed similar improvements in glycemic parameters; however, patients carrying the minor A allele had a lower reduction in PI/I ratio compared with the GG carriers. These results suggest that exenatide could be more beneficial in homozygous wild-type allele carriers early after diagnosis of T2D.

Genetic variability in *TCF7L2* gene confers risk of T2D partially through incretin-mediated insulin secretion [68]. Ferreira et al. investigated the influence of rs7903146 variant in *TCF7L2* gene on response to treatment with exenatide. A total of 56 patients with T2D underwent a 500-calorie mixed-meal test before and after treatment with exenatide for 8 weeks. Interestingly, only carriers of the minor T allele showed a reduction in insulin levels as a response to meal test after the treatment. Authors suggested that in some aspects T allele confers better response to GLP-1RA, possibly through enhanced insulin sensitivity [98].

Recently, one pharmacogenetic study was performed to test the pharmacogenetic effect of SGLT2i and GLP-1RA dual therapy in regard to body weight loss in individuals without T2D [99]. It has been shown that gliflozins and GLP-1RA provide a sustainable decrease in body weight and glycemic parameters when combined in therapy [100]. A total of 40 obese participants without diabetes were included in the study and genotyped for seven SNPs that are known to affect the GLP-1-mediated pathway. Only the minor A allele of the rs10010131 in the wolframin (WFS1) gene was significantly associated with greater body weight loss. The A allele of the WFS1 gene was previously associated with a protective role against the development of T2D [101]. However, the study lacked monotherapy arms, thus it is difficult to make clear conclusions on the association of the tested variant and drug combination. A summary of pharmacogenetic studies of GLP-1RA is shown in Table 3.

**TABLE 3:**
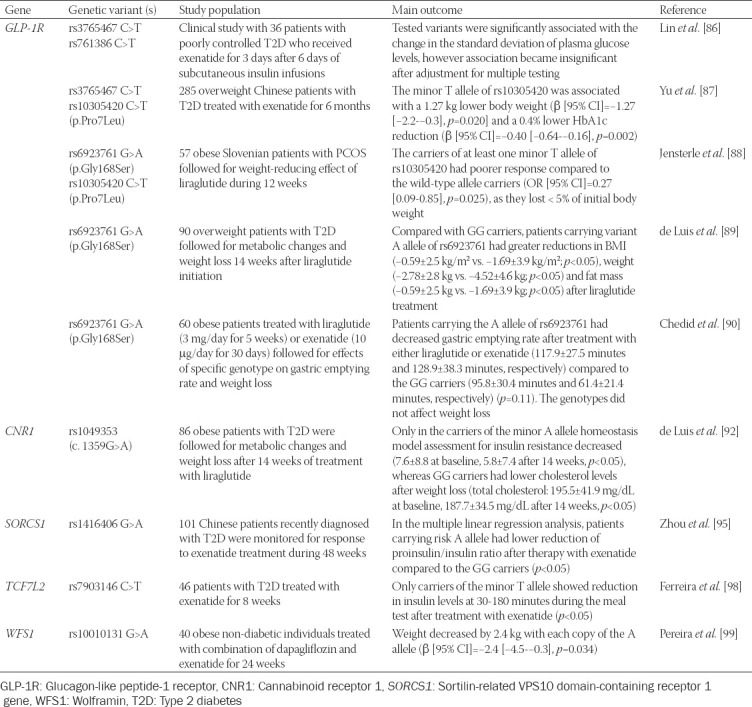
Pharmacogenetic studies of GLP-1RA

## CONCLUSION

It is evident that interest in investigation of the pharmacogenetic-based treatment continuously expands. This review presents the up-to-date knowledge of the genetic biomarkers that could influence the response to the new classes of antidiabetic drugs. A considerable amount of data has been published in recent years targeting genetics of the glycemic response to SGLT2 inhibitors, DPP-IV inhibitors, and GLP-1RA. However, it is still evident that the lack of replication is the main challenge for the pharmacogenetic studies. Furthermore, a notable number of considered studies might have had a lack of proper study designs and power due to small sample sizes, which lead to some conflicting findings. Therefore, it seems essential to gain more information from larger controlled studies to confirm the present findings. Furthermore, it might be rational to conduct a meta-analysis to aggregate the effect of the variants that appeared in multiple independent studies. Finally, it might be worth considering the effects of possible gene-gene and drug-drug-gene interactions as well as other non-genetic determinants of response to these drug classes. This should be of great interest to scientists and clinicians since these three drug classes are the preferential treatment for T2D. Further larger studies would enable us to come closer to implementing personalized treatment of patients with T2D and to significantly improve clinical outcomes.
